# Rare Pathogenic Copy Number Variation in the 16p11.2 (BP4–BP5) Region Associated with Neurodevelopmental and Neuropsychiatric Disorders: A Review of the Literature

**DOI:** 10.3390/ijerph17249253

**Published:** 2020-12-10

**Authors:** Natália Oliva-Teles, Maria Chiara de Stefano, Louise Gallagher, Severin Rakic, Paula Jorge, Goran Cuturilo, Silvana Markovska-Simoska, Isabella Borg, Jeanne Wolstencroft, Zeynep Tümer, Adrian J. Harwood, Yllka Kodra, David Skuse

**Affiliations:** 1 Centro de Genética Médica Doutor Jacinto Magalhães/Centro Hospitalar Universitário do Porto, 4099-001 Porto, Portugal; paula.jorge@chporto.min-saude.pt; 2 Unit for Multidisciplinary Research in Biomedicine, Institute of Biomedical Sciences Abel Salazar, University of Porto (UMIB/ICBAS/UP), 4050-313 Porto, Portugal; 3 Italian National Transplant Center, Italian National Institute of Health, 00161 Rome, Italy; mariachiara.destefano@iss.it; 4 Trinity Institute of Neurosciences, Trinity College Dublin, University of Dublin, 152-160 Dublin, Ireland; lgallagh@tcd.ie; 5 Public Health Institute of Republic of Srpska, 78000 Banja Luka, Bosnia and Herzegovina; severinrakic@teol.net; 6 Faculty of Medicine, University of Belgrade, 11000 Belgrade, Serbia; goran.cuturilo@udk.bg.ac.rs; 7 Department of Medical Genetics, University Children’s Hospital, Tirsova 10, 11000 Belgrade, Serbia; 8 Macedonian Academy of Sciences and Arts, 1000 Skopje, North Macedonia; silvana@manu.edu.mk; 9 Department of Pathology, Faculty of Medicine and Surgery, University of Malta, MSD 2080 Msida, Malta; isabella.borg@um.edu.mt; 10 Medical Genetics Unit, Mater Dei Hospital, MSD 2090 L-Imsida, Malta; 11 UCL Great Ormond Street Institute of Child Health, University College London, London WC1N 1EH, UK; j.wolstencroft@ucl.ac.uk (J.W.); d.skuse@ucl.ac.uk (D.S.); 12 Kennedy Center, Department of Clinical Genetics, Copenhagen University Hospital, Rigshospitalet, 2100 Copenhangen, Denmark; zeynep.tumer@regionh.dk; 13 Department of Clinical Medicine, Faculty of Health and Medical Sciences, University of Copenhagen, 2200 Copenhagen, Denmark; 14 Neuroscience and Mental Health Research Institute (NMHRI), & School of Biosciences, Cardiff University, Cardiff CF24 4HQ, UK; harwoodaj@cardiff.ac.uk; 15 National Centre for Rare Diseases, Istituto Superiore di Sanità, 00161 Rome, Italy; yllka.kodra@iss.it

**Keywords:** 16p11.2 deletion, 16p11.2 duplication, BP4–BP5, copy numbers variants, neurodevelopmental disorders, rare diseases

## Abstract

Copy number variants (CNVs) play an important role in the genetic underpinnings of neuropsychiatric/neurodevelopmental disorders. The chromosomal region 16p11.2 (BP4–BP5) harbours both deletions and duplications that are associated in carriers with neurodevelopmental and neuropsychiatric conditions as well as several rare disorders including congenital malformation syndromes. The aim of this article is to provide a review of the current knowledge of the diverse neurodevelopmental disorders (NDD) associated with 16p11.2 deletions and duplications reported in published cohorts. A literature review was conducted using the PubMed/MEDLINE electronic database limited to papers published in English between 1 January 2010 and 31 July 2020, describing 16p11.2 deletions and duplications carriers’ cohorts. Twelve articles meeting inclusion criteria were reviewed from the 75 articles identified by the search. Of these twelve papers, eight described both deletions and duplications, three described deletions only and one described duplications only. This study highlights the heterogeneity of NDD descriptions of the selected cohorts and inconsistencies concerning accuracy of data reporting.

## 1. Introduction

Copy-number variation (CNVs) is a type of structural variant involving alterations in the number of copies of specific DNA sequences, which can be deleted and/or duplicated. CNVs vary considerably in size, gene content and prevalence [1]. Although often benign, a major advance in our understanding is that CNVs contribute significantly to risk for several neurodevelopmental and neuropsychiatric conditions, rare diseases and congenital malformation syndromes. However, variable penetrance and expression, syndromes, as well as pleiotropy, is reported [2]. Improved understanding of these risks has implications for more accurate and timely communication of the associated clinical risks. 

The 16p11.2 region encompasses several distinct CNVs, responsible for five rare disorders identified as ORPHA entities in the Orphanet portal for rare diseases and orphan drugs [3] (Table 1). CNVs in the 16p11.2 region are associated with exchange of chromosomal material in regions of repetitive DNA sequence [4]. The most common CNV is a recurrent interstitial deletion of ∼600 kb, defined by breakpoints 4 and 5 (BP4–BP5) containing 26 known genes, four of which are OMIM morbid genes; most of the genes within this region are expressed in different regions of the brain (ORPHA:261197). 

These CNVs (deletion and/or duplication) arise with a frequency of about 3/10.000 (0.03%) [5]. This specific BP4–BP5 deletion has a population prevalence of approximately 1/2.000 (0.05%) and 0.5% among those with autism spectrum disorders (ASD) [5,6,7]. While about 71% of 16p11.2 deletions are de novo, ~70% of 16p11.2 duplications are inherited [8]; 16p11.2 duplications have been estimated to occur in about 3 in 10.000 people and are present in about 4 in 10.000 people who have mental health problems or difficulties with speech and language [9]. It is one of the most frequent single causes of neurodevelopmental disorders (NDDs) and autism spectrum disorders [10].

Several studies have described carriers of 16p11.2 rearrangements associated with increased risk of developmental/psychomotor delay, intellectual disability, ASD, obsessive and repetitive behaviours, other behavioural problems, and schizophrenia [8,9,11,12,13,14]. These clinical findings are also linked to a large number of patients with rare diseases [15]. Dysmorphic facial features and major malformations have also been observed, particularly in those 16p11.2 microduplications syndromal rare diseases [16]. 16p11.2 rearrangements may be overlooked by clinicians as they have variable penetrance and pleiotropic effects [2]. Their detection, however, may identify important genetic causes of neurodevelopmental and neuropsychiatric presentation that are useful in a clinical genetics context to inform genetic counselling and more broadly to inform clinical care for deletion carriers. Therefore, we need a better understanding of the neurodevelopmental and neuropsychiatric outcomes for 16p11.2 carriers to support evidence-based clinical care.

**Figure 1:**
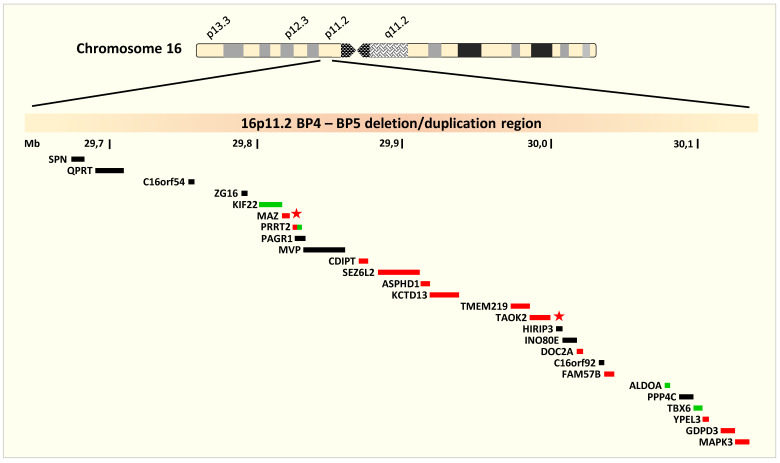
16p11.2 deletion/duplication region (GRC37/hg19 chr16:29,641,983-30,180,793). The ideogram highlighting the deletion/duplication region of interest on the short arm of chromosome 16 and the genes encompassed by BP4 and BP5. The green bars represent the OMIM morbid genes and the red bars represent the genes expressed in the brain according to GTEx data. PRRT2 (red-green bar) is an OMIM morbid gene, expressed in the brain. MAZ and TAOK2 (marked with an asterisk) are two extremely loss-of-function intolerant genes.

The BP4–BP5 region contains 26 UCSC genes (a set of gene predictions based on data from RefSeq, GenBank, CCDS, Rfam, and the tRNA Genes track, and includes both protein-coding genes and non-coding RNA genes) all of which are protein coding (Figure 1). Four genes are OMIM morbid genes [17]: *KIF22* associated with autosomal dominant spondyloepimetaphyseal dysplasia with joint laxity, type 2 (OMIM 603546); *ALDOA* associated with autosomal recessive glycogen storage disease XII (OMIM 611881), and *TBX6* associated with autosomal dominant and recessive Spondylocostal dysostosis 5 (OMIM 122600). The last gene *PRRT2* is associated with three distinct autosomal dominant disorders, familial infantile convulsions with paroxysmal choreoathetosis (OMIM 602066), episodic kinesigenic dyskinesia 1 (OMIM 128200) and benign familial infantile seizures 2 (OMIM 605751). According to GTEx expression data, most of the genes, except for a few such as *SPN, C16orf54* and *ZG16*, are expressed (though in different levels) in different regions of the brain [18]. Notably, *CDIPT*, *SEZ6L2, ASPHD1, DOC2A*, *FAM57B* and the OMIM morbid gene *PRRT2*, which is associated with seizures and dyskinesia, are highly expressed in different brain regions and some of them are highly brain specific. Genes *MAZ* and *TAOK2*, which are also brain expressed genes, are classified as “extremely loss-of-function intolerant genes” according to the computational pLI score [19].

This article provides a review of the current understanding of neurodevelopmental and neuropsychiatric phenotypes described in the cohorts with 16p11.2 (BP4–BP5) CNVs, involving deletions and/or duplications. The aim is to clarify the reported frequency of neurodevelopmental or neuropsychiatric phenotypes in the literature to date, to identify inconsistencies in phenotype descriptions that may improve or standardize the approach in the future.

## 2. Materials and Methods

### 2.1. Searching Strategy

The literature review was conducted using the PubMed/MEDLINE electronic database. The search was limited to English language papers published between 1 January 2010 and 31 July 2020. Descriptors used for the search were “16p11.2”, “Schizophrenia”, “Intellectual disabilities”, “Mental retardation”, “Intellectual disabilities”, “Autism spectrum disorders”, “Attention deficit hyperactivity disorder”, “Communication disorders”, “Specific learning disorders” and “Motor disorders” in the title and/or abstract of all papers. The Boolean operators used were “AND” and “OR”. 

### 2.2. Study Selection: Inclusion and Exclusion Criteria

Regarding the inclusion criteria, we selected only studies based on patients’ cohorts and referring to cross-sectional, prospective or retrospective studies (minimum of five carriers/patients described); subjects in the cohort had molecular confirmation of 16p11.2 BP4–BP5 microdeletions and microduplications (e.g., through FISH, MLPA, or microarray/aCGH analysis); neurodevelopmental and/or neuropsychiatric phenotypes/outcomes as described by search descriptors were reported either in the paper or, alternatively, in the paper’s tables. 

Exclusion criteria were: papers regarding non neurodevelopmental and/or neuropsychiatric phenotypes/outcomes (e.g., head size, obesity); papers reporting neuroimaging findings and systematic reviews or meta-analyses papers.

All articles were screened by title and abstracts to identify those that fitted the inclusion criteria. Next, the full text of the paper was evaluated. The papers were assessed independently for inclusion by two or three authors and imported to Mendeley, a free web-based manager for organizing references; any duplicates were excluded (Table 2 and Table 3). 

### 2.3. Data Extraction

The following information was extracted from the selected articles: (1) study identification (author, year); (2) ascertainment (16p11.2); (3) age range of subjects (years); (4) sample size; (5) the proportion or percentage of 16p11.2 deletions and/or duplications with neurodevelopmental or neuropsychiatric phenotypes according to the following domains: cognition, speech and language problems, NDDs, mood disorders, seizures and behaviour problems. The neurodevelopmental or neuropsychiatric phenotypes phenotypic data for 16p11.2 (BP4–BP5) microdeletions and microduplications are presented separately (Table 2 and Table 3). 

## 3. Results

### 3.1. Review of the Literature

A total of 75 articles were identified and screened for inclusion. Sixty-three papers were excluded because they used population cohorts, the genetic diagnosis was not clinically confirmed, the studies did not include NDD outcomes, they did not report on 16p11.2 (BP4–BP5) or they were review or meta-analysis articles about 16p11.2.

The 12 selected articles included eight papers describing phenotypes for both deletions and duplications, three reporting phenotypes for deletions only and one for duplications only. Two tables were constructed to support this review of phenotypic features (noted frequency, rate) included in the articles. Table 2 and Table 3 describe frequency of phenotypic outcomes for the 16p11.2 microdeletions (nine articles) 16p11.2 microduplications (seven articles), respectively.

### 3.2. Proximal Deletions

Nine papers reported neurodevelopmental or neuropsychiatric outcomes for microdeletions (Table 2). The selected papers described phenotypes in children, adolescents and adults of both male and female genders ranging in age from five months to fifty-nine years. Males were overrepresented among all carriers with the exception of one study where the CNV was preferentially transmitted from mothers [4]. This was the only study noting transmission data.

Probands were determined using a variety of methods, which differed between studies. These included identification through clinical genetic testing, on the basis of having a neurodevelopmental disorder or as part of cascade screening in families where a prior carrier was identified. Clinical phenotypes were reported based on either direct assessment or clinical reports.

Cognitive functioning was described to be impaired in carriers in all studies but the proportion of those affected varied. Three smaller studies reported intellectual disability in almost all of the probands [14,20,21]. Other studies noted ID in 10–26% of probands [4,13,22,23]. One study did not report cognitive ability [8], and one other only stated the range of IQ for probands [24].

Impairments in speech and language functioning have previously been highlighted in 16p11.2 carriers. Of the nine studies reviewed here speech and language delay, non-verbal language status and articulation difficulties were reported variably between studies. The proportion affected was addressed in six studies, indicating speech and language difficulties in 54–100% of probands [4,13,14,20,21,22].

Reported neurodevelopmental disorders included autism diagnosis or autism features, ADHD/ADHD features and psychosis (including schizophrenia). Autism and autism features were described in all studies and the range varied between 5–100% of cases [4,8,13,14,20,21,22,23,24]. ADHD or ADHD features were reported in six studies ranging from 19–60% [14,20,21,22,23]. Schizophrenia was not found in any of the probands in five studies [8,20,21,23,24] and was not noted in four studies [4,13,14,22].

Four studies investigated the presence of seizures in probands. One study found no evidence of seizures [20]. D’Angelo et al. 2016 [8] noted seizures in 22% of cases, while Zufferey et al. 2012 [4] and Shinawi et al. 2010 [16] reported seizures in 24% and 30% of cases respectively. 

Other psychiatric and behavioural disorders were also communicated. Oppositional defiant disorder (ODD) and aggression were reported in six studies with rates ranging from 0% to 13%. Depression, anxiety disorders and phobias were also described in a small number of studies; however, the proportions of cases with these diagnoses were low and appeared comparable with rates in the general population. 

### 3.3. Proximal Duplications

Seven studies described phenotypes in subjects with 16p11.2 proximal duplications [8,14,20,21,22,23,25]. Only one of the studies did not overlap with others reporting phenotypes in proximal deletions [25]. The cohorts in these studies included children, adolescents (ranging from one to 17 years) and adults; one study reported only the participants mean age; it included adults with a mean age of 24.2 years [8]. Male carriers were overrepresented in all cohorts with one exception [20].

Cognitive functioning and/or evidence of developmental delay in carriers was described in five studies; the rates varied widely. Developmental regression was reported in three studies; it was detected in a small number of individuals. Speech and language delay ranged from 26–86% in four studies. Two further studies reported articulation difficulties in 22% [25] and 35% of cases [22].

The presence or absence of seizures was noted in four studies and rates ranged from none to 19.4% of cases [8,14,20,21].

The most common described neurodevelopmental disorder was autism spectrum disorder, which was found in 20–27% of cases in four larger studies [8,22,23,25]. Fernandez et al. 2010 reported ASD or autism traits as an outcome in three observed cases [20]. Rosenfeld et al. 2010 found one case of ASD among seven carriers [21] and Shinawi et al. 2010 found evidence of autism traits in one of seven carriers [16]. Schizophrenia was not noted as an outcome in any study. ADHD or ADHD traits were described in five studies. ADHD was reported in 26–44% of cases. One study also highlighted Developmental Coordination Disorder in 54% of cases.

The prevalence of behavioural problems ranged from 13% to 30%, and the proportion found to have mood and/or anxiety disorders also varied substantially between studies. Anxiety disorders were more common (4–17.4%) [25], while only a small number of individuals were reported to have depression or OCD.

## 4. Discussion

The objective of this review was to examine recently reported neurodevelopmental and neuropsychiatric outcomes associated with a 16p11.2 proximal (BP4–BP5) microdeletion or microduplication. These two CNV syndromes are associated with intellectual disability (ID) and developmental delay (DD) or neuropsychiatric presentations, particularly ASD [23], [26], other neurological, behaviour and mental disorders. In brief, our review of these recent studies is consistent with the emerging view that a substantial proportion of carriers of both the 16p11.2 proximal deletions and the proximal duplications are affected by ID/DD and neurodevelopmental disorders (NDD), particularly ASD and ADHD.

Intellectual disability or developmental delay (ID/DD) affected carriers of both deletions and duplications, and may occur more frequently in carriers of the proximal deletion; ID/DD was reported in approximately 70% of deletion cases compared to approximately 35% in those with duplications. Many carriers of both deletions and duplications had a history of speech and language delay, but there was greater variability among those with proximal duplications. 

Neurodevelopmental disorders were observed to affect between 20% and 30% of carriers with deletions and duplications. In the deletion carriers there was significant variability in the proportion with ASD or autism traits between studies. Larger studies tended to find higher rates of ASD or autism traits, but used patient collections that had systematically screened for ASD such as the Simons VIP consortium/Searchlight [26], the ECHO study, the 16p11.2 European Consortium and the IMAGINE-ID study [8,27]. The was a relatively high prevalence of ADHD in both the deletion and duplication carriers, with a higher rate in those with duplications. Duplication carriers have previously been shown to have more than double the risk of ADHD compared with deletion carriers [23].

The prevalence of major psychiatric disorders (psychosis and schizophrenia) was not elevated in any of the cohorts reviewed here [23], contrasting with previous reports of psychotic symptoms in association with 16p11.2 duplications [28,29]. A recent meta-analysis of 36.676 schizophrenia patients and 48.331 healthy controls from 24 independent samples showed a significantly increased odds of developing schizophrenia in carriers of the 16p11.2 microduplication [30]. In contrast, a study of 217 deletion carriers, 114 duplication carriers, and family-based controls did not identify cases with schizophrenia beyond four who had been ascertained on the basis of schizophrenia diagnosis [23]. The absence of psychosis or schizophrenia among the studies reviewed here may reflect the relatively young mean age of the cohorts, who were yet to enter the age range usually associated with highest risk of emerging symptoms. Although many had an Autism Spectrum Disorder, the risks of psychosis are independent of autism in 16p11.2 duplication carriers [2].

We found evidence of an increased risk of recurrent seizures in association with 16p11.2 CNV. Shinawi et al. reported a series of 16p11.2 deletion and duplication patients; 3/10 duplication patients had seizures, one of which was associated with a de novo rearrangement [16]. Bijlsma et al. reported three patients with 16p11.2 deletions and history of developmental delay and seizures, of which one was a de novo rearrangement [31]. Kumar et al. also reported one ASD patient with a 16p11.2 deletion and history of seizures [11]. Ghebranious et al. described a 16p11.2 microdeletion in monozygotic twins with complex phenotypes that included seizure disorder with onset at 11.5 and 13 years of age, along with mental retardation and heart defects [32]. It appears from this review that vulnerability to seizures affects approximately 20% of both deletion and duplication CNV carriers, a proportion that is similar to that associated with ID/DD and ASD of diverse genetic aetiology, suggesting that the association with 16p11.2 is non-specific. 

Behavioural phenotypes are characteristically found among individuals with pathogenic CNV. These include problems, as defined by the Human Phenotype Ontology (HPO) [33], that encompass abnormalities of mental functioning including various affective, behavioural, cognitive and perceptual abnormalities. In other words, a behavioural phenotype is a characteristic pattern of social, linguistic, cognitive and motor observations consistently associated with a genetic disorder. In general, these problems were infrequent, mostly around 10% of cases in both CNV types, and vary between reports. Their presentation is not static and typically varies according to the level of learning disability and a host of environmental, developmental and therapeutic influences; it changes with increasing age. 

Finally, 16p11.2 deletion and duplication carriers have also been considered to confer an increased risk of other mental health problems including anxiety and depression. In our reviewed studies, a small number of individuals with microdeletions or duplications exhibited other mental health problems, such as anxiety, depressions, specific phobia or Obsessive-Compulsive Disorder (OCD). We found that frequency of anxiety, depression and phobias are almost the same (8-12%) in all the samples and both CNV types, as reported in Zufferey et al. 2012 [4], Hanson et al. 2015 [14] and Niarchou et al. 2019 [23]. 

In summary, these reports show the strongest association between the presence of a 16p11.2 CNV and ID/DD and ASD. For the 16p11.2 microdeletion, this appears to present in ID/DD in approximately 70% of carriers, dropping to approximately 30% for ID/DD in duplications and for NDD for both CNV types. Further studies will be required to confirm whether the relatively high frequency of ID/DD in association with the microdeletion is a consistent feature. Although other psychiatric, behavioural and mood disorders have also been reported, neither their frequency nor association with the CNV carriers is strong. An increased risk of recurrent seizures occurs; however, it is no greater than expected within a sample of young people with ID/DD and ASD of genetic aetiology. 

### Future Perspectives

Our review of these studies emphasises that there is a wide range of phenotypic variation and differences in severity of the clinical outcomes associated with both deletions and duplications of 16p11.2. This aligns with the previously reported variable penetrance of 31% and 34% for 16p11.2 deletions and duplications, respectively [34]. There may, however, be several factors leading to variation in clinical presentation, some of which may be reduced or eliminated, through experimental design and standardisation; others are inherent to these syndromes and will need to be accommodated by our analytical approaches.

A significant concern will always be variation and bias in patient ascertainment and clinical assessment. Most reports reviewed here used subjects that had been recruited from the Simons VIP Consortium, thus ensuring that patient data had been collected through a common protocol and in accordance with recognized data standards [26]. In addition, this recruitment was genetics-led; participants were recruited primarily because they possessed a 16p11.2 CNV potentially preceding a standardised clinical assessment. 

Conversely, in a few other reports the phenotyping methodology that followed clinical referral is not detailed and some cases considered “not seriously affected” by ID or behavioural characteristics may have been excluded from further assessment. This exclusion would have lead to ascertainment bias, which is more likely in smaller studies and should be considered when comparisons are made between reports or prevalence. Furthermore, prior selection of patient groups based upon particular diagnostic criteria such as ASD, could lead to the overrepresentation of diagnostic sub-groups of the CNV cohorts. 

We conclude that it is possible to draw high-level inferences by pooling data, bearing in mind that differences in ascertainment, recruitment and phenotyping methodologies will impact on the depth of phenotypic analysis that can be achieved with pooled data.

We recommend the future development of innovative and standard tools to detect, annotate and interpret CNVs and to improve and standardize the phenotype descriptions of cohorts carrying the CNVs. A step forward would be to use standard ontologies for phenotypic description such as HPO [33] and NCIt [35]. Using standard ways of describing syndromal rare diseases will help to clarify diagnostic enquiries and permit the identification of similar patients in different cohorts. Standardisation is essential for clinical diagnosis, and for identifying disease-causing genes. All data from cohort studies will need to adhere to the FAIR (Findable, Accessible, Interoperable and Reusable) principles and should promote the prospective collection of standardised phenotypic data for epidemiological and clinical research. Future studies need to ensure that collected data and bioresources are compatible with large-scale objectives to compile and share data for rare NDD patients, in line with Elixir recommendations. Rare diseases cohorts, such as 16p11.2 carriers can strongly contribute to these large-scale objectives.

Beyond the goals of reducing ascertainment bias, establishing standard clinical assessments and the creation of interoperable datasets, there is a need better to understand the origins of variability within sample populations due to genetic and environmental factors. Family background factors have been shown to influence cognitive ability, social behaviour and neuromotor performance in 16p11.2 deletion carriers, suggesting that environmental factors and polygenic genetic influences affect outcomes [36]. 

The most immediately addressable factor with current technology and practice is that introduced by genetic variation. Several evidence-based data reported in the literature are consistent with the presence of additional rare (<0.1% frequency in control individuals) or larger CNVs as modifiers of disorder severity [37,38,39]. In fact, genetic variation and dosage imbalance at other loci could contribute to the observed phenotypic heterogeneity, resulting in an additive or synergistic effect on neurodevelopmental pathways and disease outcomes. A potential explanation for variable expressivity associated with microdeletion/microduplication 16p11.2 syndromes hass been proposed. This is the “two-hit” model, in which the compound effect of a relatively small number of rare variants of large effect contribute to the phenotypic heterogeneity [38,39]. The consideration of secondary mutational hits modifying the phenotypic expression of 16p11.2 CNV would entail further exome and sequencing analysis of all patients. We aknowledge that this will increase the complexity of future patient studies and accentuate the need for larger-scale investigations with the appropriate resources.

A parallel approach is to integrate clinical data with the analysis of animal and cell models of disease, although such studies are not without limitations. Both animal and cell studies provide information on the underlying biological mechanisms that may lead to patient pathology, but no one model can capture all aspects of the patient condition. A 0.44 Mb syntenic region of mouse chromosome 7 has been used to generate animal models that simulate 16p11.2 deletions and duplications. They are associated with reciprocal differences in brain structure and behaviour [40]. Mice are maintained in standardized conditions and bred to have a similar genetic background, so the phenotypic consequences of these manipulations are not readily generalizable. Use of patient-induced pluripotent stem cells (iPSC) can capture genetic diversity [41] but as yet, neither the numbers of lines that are available for study nor the depth of phenotypic analysis can explain the variation in clinical phenotypes seen in patient populations [42]. CrispR-cas9 technique can be used to generate both deletions and duplications, equivalent to those of patients [43] in human iPSC. In future, it may be possible to introduce 16p11.2 CNVs into human iPSCs that have different genetic backgrounds chosen to reflect different levels of genetic risk for the specific symptoms described above. Such an approach could allow a systematic analysis of the genetic interactions that lead to some of the phenotypic variability.

## 5. Conclusions

We reviewed research into neurodevelopmental and neuropsychiatric phenotypes reported in patient cohorts with 16p11.2 deletions and/or duplications. We have highlighted the heterogeneity of neurodevelopment disorders in clinical descriptions of the published cohorts, drawing attention to inconsistencies concerning the accuracy of data reporting. Our conclusions support the emerging view that carriers of 16p11.2 proximal deletions and duplications have high rates of intellectual disabilities and developmental delays, particularly autism spectrum disorders. The wide range of phenotypic variation and differences in severity of the clinical outcomes that were reported could be explained by inherent characteristics of the syndromes or related to the variability of research designs.

There is a need for good standardization of assessments and encoding of data. This can only be achieved through cooperation and collaboration within the global research community. International consortia and researcher networks, such as *MINDDS* [COST Action CA16210: Maximizing Impact of Research in Neurodevelopmental Disorders], are emerging to promote cooperation. Such groups are facilitating agreement and adoption of standardised methodologies, protocols and assessments, enhancing our ability for data pooling and interoperability. Working at the international level will enable researchers to build larger cohorts with good geographical coverage, and overcome the problem that no single country can provide adequate sample sizes for appropriate statistical power.

## Figures and Tables

**Table 1:** 16p11.2 CNV syndromes reported in Orphanet.

Syndrome Name	ORPHA ID	Chromosomal Location
16p11.2p12.2 microdeletion syndrome	261211	Del(16)(p11.2p12.2)
16p11.2p12.2 microduplication syndrome	261204	Dup(16)(p11.2p12.2)
Distal 16p11.2 microdeletion syndrome	261222	Distal del(16)(p11.2)
Proximal 16p11.2 microdeletion syndrome	261197	Proximal del(16)(p11.2)
Proximal 16p11.2 microduplication syndrome	370079	Proximal dup(16)(p11.2)

**Table 2:** Neurodevelopmental features in probands reported in association with proximal 16p11.2 (BP4–BP5) microdeletions.

Data Source	Ascertainment (16p11.2)	Age Range	Gender (M/F)	Cognition	Speech and Language Problems			Neurodevelopmental Disorders				Psychosis		Seizures	Behavioural Problems	Other Mental Disorders				
				ID/DD	Delay	Nonvverbal	Articulation	ASD Diagnosis	ASD Features	ADHD	AD Features	Schizophrenia	Other Features		ODD, Aggression	Anxiety	Depression	Obsessions	Phobias	Other
Fernandez et al. 2010	Diagnosis of ASD	5–18 y	2/1	3/3	2/3	1/3	N/P	3/3	0/3	1/3	0/3	0/3	0/3	N/P	0/3	1/3	1/3	0/3	0/3	0/3
Rosenfeld et al. 2010	Developmental delays, ASD, dysmorphism, congenital anomalies, seizures	0.4–20 y	8/6	13/14	9/14	2/14	1/14 ^(i)^	0/14	7/14	0/14	7/14	0/14	0/14 ^(j)^	0/14 ^(k)^	5/14	2/14	1/14	1/14	2/14	2/14
Shinawi et al. 2010	Diverse reasons for referral	0.6–9 y	10/5	13/13	15/15	1/13	N/P	2/10	2/10	4/10	1/10	N/P	N/P	5/15 ^(l)^	2/10	N/P	N/P	N/P	N/P	N/P
Zufferey et al. 2012	Simons VIP consortium + 16p11.2 European Consortium	10.7 ^(a)^	56/28	About 20%of carriers meeting criteria for ID (Intelligence-2SD)	History of speech therapy in 83% of all carriers (n = 285)	N/P	N/P	8/55 (15%) ^(e)^	N/P	N/P	N/P	N/P	N/P	24%	51/70 ^(g)^					
Hanson et al. 2015	Simons VIP consortium + Cascade testing	3–17 y	78 ^(d)^	8/78 (ID,10%) 10/78 (DD,13%)	44 (56%)	N/P	36/78 (46%)	20/78 (26%)	N/P	15/78 (19%)	N/P	N/P	Tic disorder 5(6%), Learning disorders 10/78 (13%)	N/P	10/78 (13%)	5/78 (6%)	N/P	N/P	N/P	N/P
Hyppolyte et al. 2016	Mostly European 16p11.2 cohort + Cascade testing + Estonian and replication cases from SimonsVIP consortium	4.8–59 y	35/27	Range reported only	Range reported (phonological skills, lexical skills, written language, and comprehension and verbal skills)			3/62	N/P	N/P	N/P	0/62	N/P	N/P	N/P	N/P	N/P	N/P	N/P	N/P
D’Angelo et al. 2016	Simons VIP consortium + 16p11.2 European Consortium	7.6 (4.9) ^(b)^ 16.5 (15.9) ^(c)^	187/130	N/P	N/P	N/P	N/P	51/317	N/P	N/P	N/P	0/317	N/P	69/317 (21.8%)	157/317 ^(h)^					
Bernier et al. 2017	SimonsVIP consortium	0,5-5y	18/15	5/33 (15%)	18/33(54%)	N/P	22/33 (67%)	8/33 (24%)	N/P	8/33 (24%)	N/P	N/P	2/33 (67%) ^(f)^	N/P	3/33 (9%)	2/33 (6%)	N/P	N/P	N/P	N/P
Niarchou et al. 2019	Simons VIP consortium + 16p11.2 European consortium	4.8–17.7 y	N/P	51/193	N/P	N/P	N/P	39/193	N/P	59/193	N/P	0/193	9/193	N/P	13/193	18/193	0/193	2/193	17/193	1/193
Hudac et al. 2020	Simons VIP consortium + cascade testing	0.8–20.8 y	46/43	Mean and SD reportedonly	N/P	N/P	N/P	21/89 (23.6%)	Mean and SD reportedonly	N/P	N/P	N/P	N/P	N/P	N/P	N/P	N/P	N/P	N/P	N/P
Kim et al. 2020	Simons Searchlight	2–23 y	53/57	N/P	63-84% ^(m)^	N/P	N/P	26/110 (23.6%)	N/P	N/P	N/P	N/P	N/P	N/P	N/P	N/P	N/P	N/P	N/P	N/P

N/P—not provided; y-years; M-male; F-female; ^(a)^ Probands, mean age; ^(b)^ Mean age (SD) USA; ^(c)^ Mean age (SD) Europe; ^(d)^ In total; ^(e)^ Of children assessed, no adults fulfilling ASD criteria; ^(f)^ Developmental coordination disorder; ^(g)^ More than 70% of non-ASD carriers were found to have other DSM-IV-TR diagnoses: 27 including attention deficit and disruptive behaviour disorders, anxiety disorders, mood disorders, and substance-related disorders (not further specified in the article); ^(h)^ Other Behavioural and Psychiatric Features diagnoses (no ASD): including also ADHD, anxiety disorder, OCD, ODD, learning disorders, sleep disorders, motor disorders, feeding disorders, post-traumatic stress disorder, depression; ^(i)^ Regression; ^(j)^ Specific memory problem; ^(k)^ EEG abnormalities only: 1/14; ^(l)^ EEG abnormalities only: 1/15; ^(m)^ Various speech and language delays reported.

**Table 3:** Neurodevelopmental features in proband reported in association with proximal 16p11.2 (BP4–BP5) microduplications.

Data Source	Ascertainment (16p11.2)	Age Range	Gender M/F	Cognition		Speech and Language Problems			Neurodevelopmental Disorders					Psychosis		Seizures	Behavioural Problems	Other Mental Disorders				
				ID/DD	Regression	Delay	Nonverbal	Articulation	ASD Diagnosis	ASD Features	ADHD	AD Features	Other NDDs	Schizophrenia	Other Features		ODD, Aggression	Anxiety	Depression	Obsessions	Phobias	Other
Fernandez et al. 2010	All cases ascertained on the basis of a diagnosis of ASD	2.2–15.5y	1/2	1/14 (MR)	3/3	2 (1 NT)	2 (1 NT)	2/3	2/3	1/3	0/3	0/3	0/3	0/3	0/3	1/3 ^(h)^	0/3	1/3	0/3	0/3	0/3	0/3
Rosenfeld et al. 2010	Developmental delays, ASD, dysmorphism, congenitalanomalies, seizures	1–7 y	6/1	6/7	1/7^(j)^	4/7	0/7	0/7	1/7	0/7	0/7	2/7	N/P	N/P	N/P	0/7 ^(i)^	2/7	0/7	N/P	0/7	0/7	0/7
Shinawi et al. 2010	Diverse reasons for referral	4–13 y	5/2	6/7	0/7	6/7	0/7	1/7	0/7	1/7	5/7	0/7	N/P	N/P	N/P	3/7	2/7	N/P	N/P	N/P	N/P	N/P
Snyder et al. 2016	Clinical, online recruitment and laboratory referrals	1.1–17.3 y (46)	26/20	19/46 (41%)	N/P	N/P	N/P	10/46 (22%)	11/46 (24%)		12/46 (26%)	N/P	2/46 (4.4%) ^(a)^	N/P	25/46 (54%) ^(e)^	N/P	6/46 (13%)	8/46 (17.4%) ^(d)^	N/P	N/P	N/P	2/46 (4%) ^(c)^
D’Angelo et al. 2016	Simons VIP consortium + 16p11.2 European Consortium	9.1 (8.8) ^(f)^–24.2 (21.9) ^(g)^	104/7 6	N/P	N/P	N/P	N/P	N/P	36/180	N/P	N/P	N/P	N/P	4/180	N/P	35/180 (19.4%)	71/180 ^(b)^					
Bernier et al. 2017	Simons VIP consortium	0.5–5 y	13/10	5/230 (22%)	N/P	6/2 (26%)	N/P	8/2 (35%)	5/23 22%	N/P	9/23 (39%)	N/P	N/P	N/P	13/23 (56%) ^(e)^	N/P	7/23 (30%)	1/23 (4%)	N/P	N/P	N/P	N/P
Niarchou et al. 2019	Simons VIP consortium + European 16p11.2 consortium	3.1–17.3 y	N/P	31/89	N/P	N/P	N/P	N/P	24/89	N/P	39/89	N/P	N/P	0/89	8/89	N/P	11/89	10/89	0/89	2/89	8/89	2/89
Hudac et al. 2020	Simons VIP consortium	1.7–23.4 y	17/24	Mean and SD reported only	N/P	N/P	N/P	N/P	9/41 (22%)	Mean and SD reported only	N/P	N/P	N/P	N/P	N/P	N/P	N/P	N/P	N/P	N/P	N/P	N/P
Kim et al. 2020	Simons Searchlight	2–23 y	34/24	N/P	N/P	46–96 % ^(k)^	N/P	N/P	13/58 (22.4%)	N/P	N/P	N/P	N/P	N/P	N/P	N/P	N/P	N/P	N/P	N/P	N/P	N/P

MR—mental retardation; y—years; M—male; F—female; ^(a)^ Learning disorder; ^(b)^ Other Behavioural and Psychiatric Features diagnoses (NO ASD): including also ADHD, anxiety disorder, OCD, ODD, learning disorders, sleep disorders, motor disorders, feeding disorders, post-traumatic stress disorder, depression; ^(c)^ Mood disorders; ^(d)^ Anxiety/OCD/phobia; ^(e)^ developmental coordination disorder; ^(f)^ Mean age (SD); ^(g)^ mean age Europe (SD); ^(h)^ EEG abnormalities only; ^(i)^ EEG abnormalities only; ^(j)^ specific memory problem ^(k)^ Mean and SD reported only; ^(l)^ Various speech and language delays reported, not matching defined categories.
